# Non-linear longitudinal associations between moderate-to-vigorous physical activity and adiposity across the adiposity distribution during childhood and adolescence: Gateshead Millennium Study

**DOI:** 10.1038/s41366-018-0188-9

**Published:** 2018-08-14

**Authors:** Xanne Janssen, Laura Basterfield, Kathryn N. Parkinson, Mark S. Pearce, Jessica K. Reilly, Ashley J. Adamson, John J. Reilly

**Affiliations:** 1 University of Strathclyde, School of Psychological Science and Health, Glasgow, Scotland; 2 Newcastle University, Institute of Health & Society, Newcastle upon Tyne, UK; 3 Newcastle University, Human Nutrition Research Centre, Newcastle upon Tyne, UK

**Keywords:** Risk factors, Cardiovascular diseases

## Abstract

**Objective:**

Insufficient moderate-to-vigorous intensity physical activity (MVPA) is harmful for youth; however, the evidence for differential effects by weight status is limited. The study aimed to examine associations between MVPA and adiposity by weight status across childhood and adolescence.

**Methods:**

Participants were from the Gateshead Millennium Study. Physical activity and body composition measures were taken at age 7 y (*n* = 502; measures taken between October 2006 and December 2007), 9 y (*n* = 506; October 2008–September 2009), 12 y (*n* = 420; October 2011–September 2012), and 15 y (*n* = 306; September 2014–September 2015). Participants wore an ActiGraph GT1M and epochs were classified as MVPA when accelerometer counts were ≥574 counts/15 s. Weight and height were measured using standardized methods and fat mass using bioelectrical impedance. Associations between MVPA and changes in BMI and FMI were examined by weight status using quantile regression.

**Results:**

Higher MVPA was associated with lower FMI for the 25th, 50th, 75th, and 90th percentile and lower BMI at the 50th, 75th, and 90th percentile, independent of accelerometer wear time, sex, and sedentary time. The association between MVPA and change in adiposity was stronger in the higher than lower FMI and BMI percentiles (e.g., 1 h/day more MVPA was associated with a 1.5 kg/m^2^ and 2.7 kg/m^2^ lower FMI at the 50th and 90th FMI percentiles, respectively).

**Conclusions:**

The effect of MVPA on adiposity in the higher adiposity percentiles is stronger than reported to date. Given overweight and obese children are the highest risk group for later obesity, targeting MVPA might be a particularly effective obesity prevention strategy.

## Introduction

Recent studies have shown low levels of physical activity (PA) in children which decrease even further during both childhood and adolescence [1,2]. In addition, the prevalence of obesity among children and adolescents has increased to alarming levels in countries all around the world [3]. It is believed that one way to counteract this rise in obesity is via promotion of PA. However, while cross-sectional studies have shown relatively consistent positive associations between PA and adiposity, the evidence is less clear when looking at the longitudinal associations [4].

Poitras et al. showed only half of the longitudinal studies included in their review reported higher levels of moderate-to-vigorous physical activity (MVPA) were associated with lower levels of adiposity. In addition, the quality of the studies included in the review was low and most of the longitudinal studies covered a period of 2 years or less. Since then several studies examining the longitudinal association between objectively measured MVPA and adiposity have been published showing a positive association of baseline MVPA and adiposity later on [5–7]. However, two of these studies were of a relatively short duration (i.e., 2 years) and included only one follow-up time point [5, 7].

To the authors’ knowledge to date only two studies have examined changes in MVPA and adiposity over childhood and adolescence including several follow-up time points [6, 8]. Kwon et al. (2015) examined changes in PA and adiposity from age 5 to 19 years. The authors reported that those who decreased their MVPA from age 5 to 19 were at higher risk of becoming obese in adulthood compared to participants who remained active throughout. The second study looking at changes in adiposity over childhood and adolescence was conducted by Mitchell et al. [8]. Mitchell et al. [8] reported that time spent in MVPA at age 9 was associated with a change in adiposity from age 9 to 15, with a stronger association being present in participants who were at the higher end of the BMI distribution at age 9 years. This finding is especially interesting as the higher end of the BMI distribution includes participants who are often the focus in clinical practice and public health interventions. In addition to this, a more recent cross-sectional study using a quantile regression approach, supported the results by Mitchell et al. [9]. However, to the authors’ knowledge, no other longitudinal studies have used an approach which considers the possibility of associations which differ in strength, and linear regression analysis may underestimate the effect of MVPA in the higher percentiles of the adiposity distribution. Therefore, the current study will examine if MVPA is associated with changes in adiposity by weight status and compare this to whole sample linear regression analysis. We hypothesized that MVPA is associated more strongly with adiposity at the higher end of the adiposity distribution and the impact of MVPA on adiposity is underestimated using linear regression that models the mean of adiposity outcome variables.

## Methods

### Cohort

This study was conducted as part of the Gateshead Millennium Study (GMS; [10]). The GMS is a longitudinal cohort study set in Gateshead, England. For the present study measurements taken at age 7 y, 9 y, 12 y, and 15 y were included. Baseline measures for this study were taken between October 2006 and December 2007 (7 y); follow-up was conducted between October 2008 and September 2009 (9 y); between October 2011 and September 2012 (12 y), and between September 2014 and September 2015 (15 y). Child’s date of birth, sex, and parental socio-economic position, measured by Townsend score (an area-based measure derived from the UK census in 1991) were recorded at birth, all other measured used in this study were recorded at baseline and each of the follow-up time points. Written parental consent was obtained during each data collection period and the study was approved by the Gateshead and South Tyneside Local National Health Service Research Ethics Committee for data collection at 7 y and by the Newcastle University Faculty of Medical Sciences Ethics Committee for the 9 y, 12 y, and 15 y data collections.

### Body measurements

Height and weight were measured during baseline (7 y) and follow-up (9 y, 12 y, and 15 y) using standardized methods. Height was measured to the nearest 0.1 cm using a Leicester portable height measure (Chasmors, London, UK). Weight (kg) was measured and fat mass was estimated using bio-impedance while children wore light clothing using a Tanita TBF300MA. Bio-impedance data was used to first calculate age-specific and sex-specific total body water using validated prediction equations [11] after which lean mass was calculated using age-specific and sex-specific hydration factors as described by Lohman [12]. Fat mass was then calculated from weight (kg) minus lean mass. Body mass index (BMI) was calculated as weight (kg) divided by height (m) squared. Fat mass index (FMI) was calculated as fat mass (kg) divided by height (m) squared.

### Accelerometry—objective measurement of physical activity

Physical activity was measured using Actigraph GT1M accelerometers (ActiGraph Corporation; Pensacola USA) worn for 7 days during baseline and follow-up data collection points. Participants were asked to wear the accelerometer on their right hip during all waking hours, except during water-based activities. Participants recorded times the monitor was worn using a wear time diary and non-wear time and sleep time were removed based on the wear time diaries and visual inspection by a trained researcher. This method has been used in several previous studies [2, 13–15]. In addition, the consecutive zeros method to remove non-wear time was not used as it has been shown to affect the outcome significantly, especially in longitudinal studies in children and adolescence [16]. Data were collected in 15-s epochs and included in the analyses if participants had at least three days with ≥6 h per day of accelerometer data [17]. Epochs were defined as sedentary behavior (SB), light physical activity (LPA), and MVPA when recorded counts were ≤25 counts/15 s, between 25 and 573 counts/15 s and ≥ 574 counts/15 s, respectively [18].

### Statistical analysis

For descriptive purposes, the means and standard deviations for anthropometric and physical activity outcomes were calculated at each time point. Associations of time spent in MVPA with changes in BMI and FMI were examined by weight status using quantile regression analyses. A first order autogressive correlation structure was used to account for repeated measures. The 95% confidence intervals (95% CIs) were estimated using 500 cluster bootstrap samples to account for the dependence between repeated measures [19]. The interpretation of quantile regression is similar to that of linear regression with the benefit of examining different quantiles of the distribution separately. First, the effect of the independent variable (i.e., MVPA) was modeled for the 10th, 25th, 50th, 75th and 90th BMI or FMI percentile (model 1). Second wear time, sex, socio-economic status (i.e., Townsend score), and SB were added as covariates to model 1 (model 2). Time was coded as 0, 2, 5, and 8 for age 7, 9, 12, and 15 years, respectively, to enable easy interpretation of the coefficients (i.e., change in BMI/FMI per year). In addition to quantile regression, mixed effects linear regression analyses were conducted so that the impact of choice of regression approach could be compared. Mixed effects linear regression account for the correlation between repeated measures of the same participants over time. All analyses were performed using STATA 12 (StataCorp, College Station, Texas, USA).

## RESULTS

Descriptive results of each data collection point are shown in Table 1. Briefly, 502, 506, 420, and 306 participants provided valid data at age 7 y, 9 y, 12 y, and 15 y, respectively. The sample included an almost equal split of girls and boys. At age 7 y participants participated on average 69.8 min/day in MVPA. By the time participants were 15 y old, this had decreased to 46.4 min/day in MVPA. On average, time in MVPA decreased by −1.1% (SD ± 3.0) for 7 y to 9 y (equivalent to −6.1 min/day), −1.8% ( ± 3.6) for 9 y to 12 y (−9.4 min/day), −1.1% ( ± 3.2) for 12 y to 15 y (−8.5 min/day).

**Table 1:** Summary of the participants at each data collection period

		**Data collection period**			
		**7y**	**9y**	**12y**	**15y**
Sample	*n* (% with valid data)	502 (97)	506 (98)	420 (95)	306 (94)
Sex (male)	%	50.2	48.2	46.9	46.7
Age (years)	Mean (SD)	7.5 (0.5)	9.3 (0.4)	12.5 (0.3)	15.2 (0.4)
Weight (kg)	Mean (SD)	26.4 (5.2)	33.5 (7.6)	49.6 (12.2)	62.2 (14.1)
Height (cm)	Mean (SD)	124.9 (5.6)	135.6 (6.4)	154.6 (7.8)	167.0 (8.3)
BMI (kg/m^2^)	Mean (SD)	16.8 (2.3)	18.0 (2.9)	20.6 (3.9)	22.2 (4.4)
FMI (kg/m^2^)^a^	Mean (SD)	4.0 (1.8)	4.8 (2.4)	5.4 (3.3)	6.5 (4.1)
SB (min)	Mean (SD)	346.5 (66.4)	372.8 (63.3)	466.5 (86.6)	535.1 (85.6)
SB (% of wear time)	Mean (SD)	51.5 (7.7)	55.4 (7.0)	64.8 (8.2)	73.4 (6.6)
LPA (min)	Mean (SD)	254.9 (44.9)	236.8 (43.0)	197.4 (45.9)	144.1 (34.5)
LPA (% of wear time)	Mean (SD)	38.2 (5.6)	35.3 (5.2)	27.7 (6.1)	20.1 (4.8)
MVPA (min)	Mean (SD)	69.8 (24.0)	63.0 (22.9)	53.9 (23.3)	46.4 (20.0)
MVPA (% of wear time)	Mean (SD)	10.5 (2.6)	9.4 (3.2)	7.6 (3.3)	6.5 (3.0)

*LPA* time spent in light physical activity per day, *MVPA* time spent in moderate-to-vigorous physical activity per day, *BMI* body mass index, *FMI* fat mass index

^a^Smaller sample due to missing data (7 y *n* 500, 9 y *n* 424; 12 y *n* 378; 15 y *n* 298)

### MVPA and adiposity using quantile regression

**Fig. 1:**
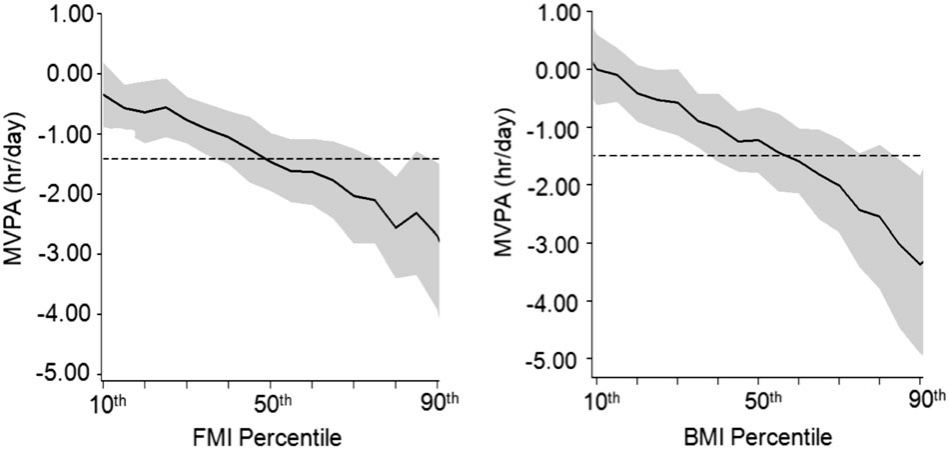
Quantile regression association between MVPA and FMI and BMI. The plots are based on model 3 which controlled for sedentary time and sex. The dotted line represents the linear regression coefficient for the change in FMI and BMI per hour MVPA

The results of the quantile regression analysis for MVPA and FMI and BMI are shown in Tables 2, 3. Higher MVPA was associated with a lower FMI for all but the 10th percentiles (*p* < 0.05). Every additional hour of daily MVPA was associated with a lower FMI of −0.56 kg/m^2^ (95%CI: −1.04, −0.07) at the 25th percentile, −1.46 kg/m^2^ (−2.04, −0.88) at the 50th percentile, −2.11 kg/m^2^ (−3.00, −1.20) at the 75th percentile, and −2.69 kg/m^2^ (−3.92, −1.46) at 90th percentile, independent of accelerometer wear time, sex, and SB. Higher MVPA was associated with a lower BMI for the 50th, 75th, and 90th BMI percentiles only. Every additional hour of daily MVPA was associated with a lower BMI of −1.22 kg/m^2^ (−1.94, −0.51), −2.43 kg/m^2^ (−3.47, −1.38) at the 75^th^, and −3.37 kg/m^2^ (−4.90, −1.83) at the 90th percentile, independent of accelerometer wear time, sex, and SB. The association between change in MVPA and change in BMI and FMI was stronger in the higher BMI and FMI percentiles compared to the lower BMI and FMI percentiles. In addition, analysis showed that the effect of MVPA was overestimated in the lower percentiles and underestimated in the higher percentiles of both BMI and FMI when using linear regression analysis (Fig. 1 and Appendix Table 1).

**Table 2:** Quantile regression models of MVPA and change in FMI percentiles from ages 7 to 15 years

	**FMI**				
	**10th percentile**	**25th percentile**	**50th percentile**	**75th percentile**	**90th percentile**
*Model 1*					
Intercept	2.97 (2.55, 3.39)	3.76 (3.32, 4.21)	5.10 (4.58, 5.61)	6.57 (5.84, 7.31)	8.84 (7.47, 10.21)
Time	−0.05 (−0.11, 0.01)	0.07 (0.02, 0.11)	0.19 (0.13, 0.25)	0.41 (0.33, 0.49)	0.56 (0.42, 0.69)
MVPA	**−0.66** **(**−**1.03,** −**0.28)**	**−0.77** **(**−**1.10,** −**0.45)**	**−1.19** **(**−**1.53,** −**0.84)**	**−1.47** **(**−**1.99,** −**0.94)**	**−2.15** **(**−**3.07,** −**1.22)**
*Model 2*					
Intercept	4.73 (2.97, 6.48)	3.70 (2.57, 4.84)	4.94 (3.34, 6.54)	6.15 (3.42, 8.88)	11.59 (7.47, 15.71)
Time	−0.03 (−0.12, 0.06)	0.10 (0.04, 0.16)	0.25 (0.16, 0.34)	0.47 (0.34, 0.60)	0.66 (0.49, 0.82)
MVPA	−0.35 (−0.87, 0.18)	−**0.56 (**−**1.04,** −**0.07)**	−**1.46 (**−**2.04,** −**0.88)**	−**2.11 (**−**3.00,** −**1.21)**	−**2.69 (**−**3.92,** −**1.46)**

Boldface indicates statistical significance (*p* < 0.05); MVPA, hours of moderate-to-vigorous physical activity per day. Model 1: describes changes of FMI over time with inclusion of MVPA as independent variable. Model 2: as Model 1 with inclusion of wear time, sedentary behavior, towns quintile and sex as covariates. Data presented are coefficients (95% confidence intervals). Time is coded 0, 2, 5, and 8 for age 7, 9, 12, and 15 years, respectively. The MVPA coefficients are the changes in FMI, at each percentile, for every additional hour spent in MVPA

**Table 3:** Quantile regression models of MVPA and change in BMI percentiles from ages 7 to 15 years

	**BMI**				
	**10th percentile**	**25th percentile**	**50th percentile**	**75th percentile**	**90th percentile**
*Model 1*					
Intercept	14.22 (13.69, 14.74)	15.62 (15.00, 16.25)	17.45 (16.82, 18.07)	19.70 (18.76, 20.64)	22.67 (21.23, 24.11)
Time	0.42 (0.35, 0.49)	0.49 (0.44, 0.54)	0.61 (0.54, 0.67)	0.77 (0.67, 0.87)	0.96 (0.80, 1.12)
MVPA	0.12 (−0.27, 0.50)	−0.36 (−0.79, 0.07)	−**0.97 (**−**1.42,** −**0.51)**	−**1.62 (**−**2.31,** −**0.94)**	−**2.40 (**−**3.39,** −**1.40)**
*Model 2*					
Intercept	13.99 (12.47, 15.52)	14.61 (13.20, 16.03)	16.44 (14.60, 18.29)	18.85 (15.67, 22.03)	22.03 (16.15, 27.92)
Time	0.46 (0.36,0.56)	0.52 (0.44, 0.61)	0.67 (0.59, 0.76)	0.89 (0.74, 1.04)	1.14 (0.92, 1.37)
MVPA	0.00 (−0.63, 0.62)	−0.52 (−1.16, 0.11)	−**1.22 (**−**1.94,** −**0.51)**	−**2.43 (**−**3.47,** −**1.38)**	−**3.37 (**−**4.90,** −**1.83)**

Boldface indicates statistical significance (*p* < 0.05); MVPA, hours of moderate-to-vigorous physical activity per day. Model 1: describes change in BMI over time with inclusion of MVPA as independent variable. Model 2: as Model 1 with inclusion of sedentary behavior, wear time, towns quintile, and sex as covariates. Data presented are coefficients (95% confidence intervals). Time is coded 0, 2, 5, and 8 for age 7, 9, 12, and 15 years, respectively. The MVPA coefficients are the changes in BMI, at each percentile, for every additional hour spent in MVPA

## DISCUSSION

### Main study findings and implications

The present study is one of the first to provide longitudinal evidence that higher levels of MVPA are associated with lower levels of adiposity during childhood and adolescence, and that the strength of these associations differs along the BMI and FMI distribution. In the present study, higher MVPA was associated with a lower FMI for all FMI percentiles but the 10th FMI percentile. However, higher MVPA was only associated with a lower BMI at the 50th, 75th, and 90th percentile. In addition, the association between MVPA and changes in adiposity were stronger at the higher quantiles of the FMI and BMI distribution. This may indicate that even small changes in MVPA can results in significant changes in BMI and FMI in those at the higher end of the FMI and BMI distribution. In addition, the results show that when using standard linear regression analysis the strength of the association between MVPA and adiposity can be either overestimated (lower quantiles of adiposity distribution) or underestimated (higher quantiles of the adiposity distribution).

There are several factors which may explain the associations between the difference in strength of the association between MVPA and changes in adiposity observed in the present study. It may be that the difference is simply due to the fact that those overweight and obese have more fat reserves and therefore are more likely to lose fat mass when engaging in physical activity. Alternatively, genetic differences could play a role. A meta-analysis conducted by Silventoinen et al. reported genetics play had a strong effect on the variation of BMI across childhood and adolescence [20]. This could indicate that those in the lower end of the adiposity spectrum are less likely to gain weight due to genetic reasons and therefore limited participation in PA does not necessarily lead to an increase in weight. Quantile regression evidence on the genetics of childhood obesity also suggests that the influence of genetic risk factors is stronger at the upper end of the distribution of adiposity [21], and there is evidence that higher levels of MVPA blunt the impact of adverse genetic risk factors [22]. Taken together with the present study, this emerging body of evidence suggests that MVPA is important for healthier energy balance regulation, particularly for those at high genetic risk of obesity and/or those at the upper end of the adiposity distribution. Last, the energy cost of physical activity has been shown to be higher in those who are obese compared to those who are normal weight indicating that an increase in MVPA in those in the higher adiposity percentiles results in a larger increase in energy expenditure and therefore will have a bigger effect on the energy balance [23].

### Comparisons with other studies

Recent systematic reviews have provided conflicting evidence on associations between objectively measured PA and adiposity, and have called for more studies with stronger designs, including more longitudinal studies [4]. While previous studies have been inconclusive, on balance the available evidence has suggested that an association between MVPA and adiposity was likely. The current study confirmed these results. However, the current study also indicated the strength of the association between MVPA and adiposity may be underestimated in most studies using whole sample linear regression analysis.

As mentioned previously, to date only two studies have included multiple follow-up points across childhood and adolescence. Due to differences in analysis, results between Kwon et al. (2015) and the current study are difficult to compare. However, similar to the current study, Kwon et al. did show an association between change in physical activity and adiposity. Kwon et al. reported an increased risk of becoming obese in those who decreased their physical activity from age 5 to 19 compared to those who remained stable. The results of studies using quantile regression to examine the association between MVPA and adiposity are very similar to those of the current study [8, 9]. Both studies by Mitchell et al. [8, 9] reported significant associations between MVPA and adiposity with the strength of the association increasing for each quantile of the adiposity distribution. In addition, when using linear regression the association in both the current study and the study by Mitchell et al. [8] showed an underestimation of the strength of the association in the higher adiposity percentiles. Consequently, if a study sample includes relatively few overweight and/or obese participants the strength of the association may be reduced or not be significant at all. However, the current study also showed that it is likely that many of the previous studies have underestimated the effect of MVPA on adiposity in populations of particular interest for intervention studies (i.e., those in the higher BMI and FMI percentiles).The present study also suggests that the influence of MVPA on adiposity may increase over time in populations where there is a secular trend to increased adiposity.

### Study strengths and weaknesses

The strengths of this study are the use of a contemporary cohort, the longitudinal design with multiple follow-up data collection points throughout childhood and adolescence and the use of quantile regression to expand analyses beyond linear regression analyses restricted to the mean of adiposity phenotypes. In addition, the study used high-quality measures for both exposure (accelerometry) and outcome (body composition) variables. The quantile regression analysis made it possible to highlight the different strengths in associations between adiposity and MVPA longitudinally, something that has only been done once previously. The study also had some limitations, families’ socio-economic status was measured at birth and this may have changed between birth and our baseline measures. Parental lifestyle may influence child’s behavior, however, unfortunately we did not capture this and could not include this as a covariate in our analysis. In addition, we did not control diet which may be a confounder. The sample size of the study has decreased by 40% over the 8 years of data collection; however a slightly higher attrition rate is not abnormal during longitudinal data analysis [6, 8]. The reduction in sample size may also have impacted generalizability of study findings. However, the current cohort remains contemporary (born between 1999 and 2000), and socio-economically representative of North-East England [10], and both of these characteristics should enhance generalizability.

## Conclusions

In this study MVPA declined from age 7 y to 15 y and lower levels of MVPA were associated with higher levels of adiposity throughout childhood and adolescence. The association between adiposity and MVPA was stronger at higher percentiles. This study indicates that the effect of MVPA on adiposity in the higher adiposity percentiles is stronger than reported to date. Consequently, given overweight and obese children are the highest risk group for later obesity, using MVPA interventions with overweight and obese children might be an even more effective obesity prevention strategy than initially thought.

## Disclaimer

The views expressed in this paper do not necessarily represent those of the funders or UKCRC.

## Electronic supplementary material

Supplement Table 1
