# Supplementary material for: Non-linear longitudinal associations between moderate-to-vigorous physical activity and adiposity across the adiposity distribution during childhood and adolescence: Gateshead Millennium Study
Source: Int J Obes (Lond). 2018 Aug 14;43(4):744–50. doi: 10.1038/s41366-018-0188-9 (PMC6484716; doi:10.1038/s41366-018-0188-9)
Supplement: Supplementary file 1 — Supplement Table 1 [file 41366_2018_188_MOESM1_ESM.docx]

Supplement Table 1. Linear regression analysis of MVPA and the change in BMI and BMI percentiles from ages 7-15 years.

|  | ***FMI*** | ***BMI*** |
| --- | --- | --- |
| *Model 1* |  |  |
| Intercept | 5.66  (5.20, 6.12) | 18.12  (17.60, 18.65) |
| Time | 0.23  (0.18, 0.28) | 0.65  (0.59, 0.71) |
| MVPA | **-1.39**  **(-1.75, -1.04)** | **-1.18**  **(-1.59, -0.77)** |
| *Model 2* |  |  |
| Intercept | 5.29  (3.89, 6.68) | 16.90  (15.50, 18.29) |
| Time | 0.28  (0.21, 0.35) | 0.72  (0.68, 0.79) |
| MVPA | **-1.35**  **(-1.88, -0.82)** | **-1.08**  **(-1.61, -0.54)** |
|  |  |  |

Boldface indicates statistical significance (p<0.05); MVPA, hours of moderate-to-vigorous physical activity per day. Model 1: describes change in FMI or BMI over time with inclusion of MVPA as independent variable. Model 2: as Model 1 with inclusion of wear time, sedentary behaviour, towns quintile and sex as covariates. Data presented are coefficients (95% confidence intervals). Time is coded 0, 2, 5 and 8 for age 7, 9, 12 and 15 years, respectively. The MVPA coefficients are the changes in FMI and BMI for every additional hour spent in MVPA.
